# Performance of component resolved microarray diagnosis in nuts allergy

**DOI:** 10.1186/2045-7022-5-S3-P33

**Published:** 2015-03-30

**Authors:** Maria Jose Goikoetxea, Carmen D'Amelio, Rubén Martínez, María Dolores Alonso, María Isabel Alvarado, Joan Bartra, Natalia Blanca, Paula Cabrera-Freitag, Blanca E García, Pedro Gamboa, María Luisa Sanz

**Affiliations:** 1 Clinica Universidad de Navarra, Pamplona, Spain; 2 Hospital de Coria, Caceres, Spain; 3 Hospital Clinic, Barcelona, Spain; 4 Hospital Infanta Leonor, Madrid, Spain; 5 Clinica Universidad de Navarra, Madrid, Spain; 6 Complejo Hospitalario de Navarra, Pamplona, Spain; 7 Hospital Basurto, Bilbao, Spain; 8 Hospital de Alcorcon, Madrid, Spain

## 

Performance of commercially available component resolved microarray has not been widely evaluated in food allergy. Nut allergy is a prevalent and increasing food allergy in which component resolved diagnosis is crucial.

In the context of a multicentric study on pollen and vegetable food allergy in Spain, we enrolled patients (n=100) who had suffered at least two episodes of immediate symptoms after ingestion of nuts (peanut, walnut or hazelnut). Skin prick test was positive for the corresponding nut. ImmunoCAP ISAC 112 was analyzed in all patients and ImmunoCAP to the available components from the studied sources were analyzed (Ara h 1, Ara h 2, Ara h 3 and Ara h 8 and Ara h 9, Jug r 1, Jug r 3, Cor a 1, Cor a 8, Cor a 9, Cor a 14) in those patients showing negative result to ISAC.

Among the 67 patients allergic to peanut 3 were sensitized to Ara h 1, 3 to Ara h 2, 1 to Ara h 3, 9 to Ara h 6, 1 to Ara h 8 and 36 to Ara h 9. Twenty-seven peanut allergic patients showed no sensitization to any components in the microarray. Among these 27 ISAC negative peanut allergic patients, 3 were sensitized to Ara h 1, 1 to Arah 2, 1 to Ara h 3, 1 to Ara h 8 and 23 to Ara h 9 by CAP.

Among the 69 patients allergic to walnut 7 were sensitized to Jug r 1, 6 to Jug r 2 and 45 to Jug r 3. Eighteen walnut allergic patient showed no sensitization to any component in the microarray. Among these 18 ISAC negative walnut allergic patients, 10 were sensitized to Jug r 3 by CAP (none to Jug r 1).

Among the 56 patients allergic to hazelnut one was sensitized to Cor a 1, 33 to Cor a 8 and 2 to Cor a 9. Twenty hazelnut allergic patient showed no sensitization to any components in the microarray. Among these 20 ISAC negative hazelnut allergic patients, 10 were sensitized to Cor a 8 by CAP (none to Cor a 1, Cor a 9 or Cor a 14).

Among the 100 patients allergic to peanut, walnut or hazelnut, 40 patients did not show specific IgE against the studied nuts/ components in ISAC.

Only a sixty per cent of the patients allergic to nuts (peanut, walnut or hazelnut) are diagnosed by ISAC. Sensitization to the lipid transfer proteins Ara h9, Jug r 3 and Cor a 8 was not detected by ISAC but it was recovered by CAP analysis.

